# Sickness absenteeism and associated factors among horticulture employees in lume district, southeast Ethiopia

**DOI:** 10.1186/s12995-015-0074-5

**Published:** 2015-08-12

**Authors:** Sebsibe Tadesse, Kamil Ebrahim, Zemichael Gizaw

**Affiliations:** Institute of Public Health, The University of Gondar, Gondar, Ethiopia; Labour and social affairs, Oromia Regional State, Addis Ababa, Ethiopia

**Keywords:** Employees, Horticulture, Sickness absenteeism, Ethiopia

## Abstract

**Background:**

Sickness absenteeism is the major occupational health problem in developing countries where the majority of working population are engaged in hazardous sectors, such as agriculture. However, there is a dearth of studies clarifying the situation in most of Subsaharan African countries, like Ethiopia. The present study determined the magnitude of sickness absenteeism and associated factors among horticulture employees in Lume District, southeast Ethiopia.

**Methods:**

An institutional-based cross-sectional study was conducted among horticulture employees in Lume District, southeast Ethiopia from March to May 2014. Stratified sampling followed by simple random sampling techniques was used to select the study participants. A pre-tested and structured questionnaire was used to collect data. Multivariable analyses were employed to see the effect of explanatory variables on dependent variable.

**Results:**

The magnitude of sickness absenteeism was 58.8 % [95 % CI: (54.9, 62.5)] in the past three months. Absence of periodic medical checkup, working for more than 48 h per week, working overtime, job dissatisfaction, and job stress were factors significantly associated with sickness absenteeism.

**Conclusions:**

In this study a relatively higher rate of sickness absenteeism was reported compared to other studies. Interventions to reduce sickness absenteeism should focus on areas, such as periodic medical checkup, monitoring work schedules, improving employees’ job satisfaction, and managing job stress.

## Background

Sickness absenteeism is the major occupational health problem in developing countries where the majority of working population are engaged in hazardous sectors, such as agriculture; causing loss of work-hours, reduced productivity and workplace disputes [1–5]. According to International Labor Organization 2008, more than 317 million accidents and diseases occurred on the job annually; and about two-thirds of them caused employees away from work for four working days resulting in economic effects and loss of labor time in both developing and developed countries [6]. In Subsaharan Africa, 42 million work-related accidents caused at least three days absence from work in which agriculture plays the major role [2]. About half million accidents caused at least four days off work in Ethiopia [7].

Horticulture investment is the most growing agriculture sector in East Africa, especially in the lowland regions of Ethiopia [8]. The industry uses many hazardous chemicals that may affect the health of employees if proper safety procedures are not followed [2, 5]. However, there is limited evidence on the magnitude, nature and role of different factors associated with sickness absenteeism among horticulture employees in Ethiopia. Moreover, many organizations do not have proper data recording and reporting systems to generate statistics that shows how cosmic the problem is at local and national levels [9]. Therefore, this study aimed to determine the magnitude of sickness absenteeism and associated factors among horticulture employees in Lume District, southeast Ethiopia. The findings of the study could provide information for safety officers, employers and policy makers to design strategies that aid to prevent the different health and safety hazards and improve employees’ attendance.

## Methods

### Study area, design and period

An institutional-based cross-sectional study was conducted to assess the magnitude of sickness absenteeism and associated factors among horticulture employees in Lume District, southeast Ethiopia, from March to May 2014. The district is located at 75 km East of Addis Ababa, the capital city of Ethiopia. According to the 2007 Ethiopian population statistics, the district has a total of 117,080 populations. Of whom, 51.4 % were males and 33 % were urban dwellers [10]. The study covered eight horticulture industries employing more than 80 % of all employed workers in the district. During the investigation, there are 5900 employees working in the industries; 62.9 % of whom were females [11].

### Participants and data collection

All horticulture employees who have worked for at least three months prior to the study period were included in the study until the required sample size was obtained. Women on maternity leave during the time of data collection were excluded from the study. Data were collected using structured interview questionnaire comprising of *workplace stress scale* developed by Marlin Company and the American Institute of Stress [12] and *generic job satisfaction scale* developed by Scott Macdonald and Peter Macintyre [13] with some modification. The questionnaire also contained detailed information on socio-demographic, behavioral and other workplace conditions.

### Sample size calculation

Epi info version 7 was used to determine the sample size required for this study by taking 5900 total population, 50 % expected proportion of sickness absenteeism, 4 % confidence limit, and 95 % confidence level. By adding 10 % non-response rate, the total sample size was 600.

### Sampling procedure

Stratified sampling followed by simple random sampling techniques was used to select the study participants. That is, the industries were stratified into three departments, namely production, technical and management department. Then, the total of 600 samples was proportionally allocated to each department. The participants were drawn from the factory’s list of workers using simple random sampling.

### Data quality control

The training of data collectors and supervisors emphasized issues such as data collection instrument, field methods, inclusion–exclusion criteria, time of interview, and record keeping. The completed questionnaires were spot-checked and reviewed on a daily basis to ensure the completeness and consistency of the data collected. The interview questionnaire was pre-tested on 40 respondents who had characteristics nearly similar to employees in Lume district in order to identify potential problem areas, unanticipated interpretations, and cultural objections to any of the questions. Based on the pre-test results, the questionnaire was adjusted contextually.

### Data management and statistical analyses

Data entered and cleaned using Epi info version 7 statistical software were analyzed on SPSS version 20. Frequency distribution, mean, standard deviation, and percentage, were employed for most variables. All independent variables were fitted separately into bivariate logistic model to evaluate the degree of association with sickness absenteeism. Then, variables with a p-value < 0.20 were exported to multivariable logistic regression model to control confounders. The odds ratio (OR) with a 95 % confidence interval (CI) was used to test the statistical significance of variables.

### Operational definitions

#### Horticulture

Organization that produces fruits, vegetables and flowers and avail on level of local and global market [8].

#### Sickness absenteeism

Self-reported employees’ absence from their normal duty for the reasons stemming from health problem in the past three months.

#### Job stress

The employee was considered as stressed with work when his/her sum of workplace stress scale score was 21 or above [12].

#### Satisfied with job

The employee was considered as satisfied with job when his/her sum of generic job satisfaction scale score was 32 or above [13].

#### Permanent employee

Any contract of employment between employee and employer concluded for an indefinite period [14].

#### Temporary employee

Any employment contract between employee and employer made for definite period [14].

#### Overtime work

An employee was considered as worked overtime when she/he had worked on average greater than or equal to 2 h more than the standard working hours per week within the past 3 months [14].

#### Cigarette smoker

An employee who was smoking one cigarette a day for at least one year [3].

#### Alcohol drinker

An employee who drink at least five drinks per week for men and two drinks per week for women for at least one year [3].

#### Khat chewer

An employee chewing Khat three times a week for at least one year [3].

#### Attendance-based incentive

An incentive provided for employee for his/her being only present at work.

### Ethical considerations

The study protocol was reviewed and approved by the Institutional Review Board of the University of Gondar via the Institute of Public Health. Permission was obtained from Lume District Labor and Social Affairs Office prior to data collection. Study participants were interviewed after informed written consent was obtained. They were also informed that their participation was voluntary and that they could withdraw from the interview at any time without consequences. The participants were assured that their responses would be treated confidentially through the use of strict coding measures.

### Literature search strategy

PubMed, Medline, and Scopus were the main databases searched for literature and bibliographic alerts were set up for additional papers to be identified whenever they cited key articles. Keywords for the literature search were identified by means of Medical Subject Headings (MeSH) thesaurus which is available through PubMed online (http://www.ncbi.nlm.nih.gov/pubmed). Some MeSH terms used were ‘horticulture’, ‘horticulture employees’, ‘horticulture workers’, ‘sickness absenteeism’, and ‘workplace factors’. Although the bibliographic search was meant to find the most up-to-date information, some old but highly influential papers were also reviewed. EndNote software (http://www.endnote.com/) was used to store citations together with their respective articles in pdf format, so that an annotated bibliography could be built and eventually cited and referenced.

## Results

### Socio-demographic characteristics

A total of 590 employees completed the questionnaire making response rate 98.3 %. Of whom 71.9 % were females. The mean age with a standard deviation of the employees was 26.9 ± 7.1. More than two-thirds, 70.8 %, of them belonged to the age group of 19–29 years. Those who attended primary education were 32.9 %. More than two-thirds, 70.5 %, served for less than five years. Regarding religion 74.9 % of the employees were Christian. The married were 44.6 %. Nearly half, 48.3 %, had a monthly salary of less than or equal to Birr 700 (Table 1).

**Table 1:** Socio-demographic characteristics of horticulture employees in Lume district, southeast Ethiopia, 2014

Variables	Number	Percent
Sex		
Male	166	28.1
Female	424	71.9
Age (in years)		
≤18	23	3.9
19-29	418	70.8
30-40	114	19.3
>40	35	5.9
Marital Status		
Single	252	42.7
Married	263	44.6
Widowed/divorced	75	12.7
Educational status		
Illiterate	186	31.5
Primary education	194	32.9
Secondary education	153	25.9
Above secondary education	57	9.7
Religion		
Christian	442	74.9
Muslim	148	25.1
Monthly salary (in Birr)		
≤700	285	48.3
701-1500	245	41.5
>1500	60	10.2
Work experiences (in years)		
<5	416	70.5
≥5	174	29.5

### Workplace characteristics

The majority, 89.2 %, of the respondents were permanent employees. Regarding hours spent on work 88.6 % of the employees had worked for ≤48 h per week. Two-thirds, 66.8 %, of them worked overtime. About three-fourths (71.7 %) were dissatisfied with their current job, and 62.7 % were stressed. The majority, 89.3 % and 85.4 %, of them had no pre-employment screening and periodic medical checkup, respectively. More than two-thirds, 69.0 %, of them didn’t receive attendance-based incentive (Table 2).

**Table 2:** Workplace characteristics of horticulture employees in Lume district, southeast Ethiopia, 2014

Variables	Number	Percent
Employment type		
Permanent	526	89.2
Temporary	64	10.8
Working hours per week		
≤48	523	88.6
>48	67	11.4
Work overtime		
Yes	394	66.8
No	196	33.2
Job satisfaction		
Satisfied	167	28.3
Dissatisfied	423	71.7
Workplace stress		
Not stressed	220	37.3
Stressed	370	62.7
Pre-employment medical		
Screening		
Yes	63	10.7
No	527	89.3
Periodic medical checkup		
Yes	86	14.6
No	504	85.4
Received attendance-based incentive		
Yes	183	31.0
No	407	69.0

### Magnitude of sickness absenteeism

The magnitude of sickness absenteeism among horticulture employees was observed to be 58.8 % [95 % CI: (54.9, 62.5)] in the past three months. Of whom 73.5 % were females. The majority, 72.6 % and 69.7 %, of them belonged to the age group of 19 to 29 years and attended primary and below education, respectively. The average frequency of absence was 1.73 [95 % CI: (1.63, 1.84)]. The mean number of lost working days per absentee was 6.63. The total of 2302 working days was lost.

### Perceived causes of sick absenteeism

**Fig. 1:**
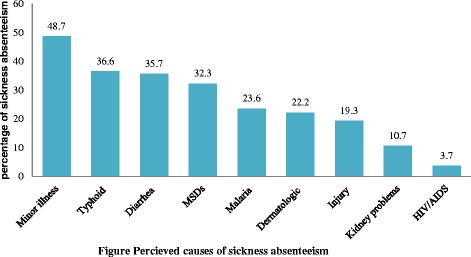
Percieved causes of sickness absenteeism

The most common health problems that lead employees away from their work were 48.7 % minor illness, 36.6 % typhoid, 35.7 % diarrhea, 32.3 % Musculo-Skeletal Disorders (MSDs) (Fig. 1).

### Factors associated with sickness absenteeism

Table 3 presents factors which remained statistically significant in the bivariate and multivariable logistic regression analyses. In this study, the independent predictors of sickness absenteeism on the multivariable analysis include absence of periodic medical checkup [AOR: 3.6, 95 % CI: (2.1, 6.1)], working for >48 h per week [AOR: 1.9, 95 % CI: (1.0, 3.5)], working overtime [AOR: 2.8, 95 % CI: (1.9, 4.2)], job dissatisfaction [AOR: 1.8, 95 % CI: (1.2, 2.7)], and job stress [AOR: 2.0, 95 % CI: (1.4, 2.9)], (Table 3).

**Table 3:** Factors associated with sickness absenteeism among horticulture employees in Lume District, southeast Ethiopia, 2014

Variables	Sickness absenteeism		Crude OR (95 % CI)	Adjusted OR (95 % CI)
	Yes	No		
Periodic medical checkup				
Yes	36	50	1	1
No	311	193	2.2(1.4, 3.6)	3.6(2.1, 6.1)
Working hours per week				
≤48	302	221	1	1
>48	45	22	1.5(0.9, 2.6)	1.9(1.0, 3.5)
Worked overtime				
No	88	108	1	1
Yes	259	135	2.4(1.7, 3.3)	2.8(1.9, 4.2)
Job satisfaction				
Satisfied	84	83	1	1
Dissatisfied	263	160	1.6(1.1, 2.3)	1.8(1.2, 2.7)
Workplace stress				
Not stressed	106	114	1	1
Stressed	241	129	2.0(1.4, 2.8)	2.0(1.4, 2.9)

## Discussion

Sickness absenteeism remains to be the major occupational health problem among horticulture employees. In this study the magnitude of sickness absenteeism among horticulture employees was 58.8 % [95 % CI: (54.9, 62.5)]. This finding is higher than that of studies from Ethiopia (53.9 %) [15], Nigeria (15.8-25.0 %) [16, 17] and Denmark (12.0 %) [18]. The difference could be due to methodological differences, like study population and methods of data collection, and workplace conditions, like employees’ level of awareness on hazard control and disease prevention and accessibility to health care services [15–18].

This study identified important predictors influencing sickness absenteeism. Employees who didn’t undergo periodic medical checkup were more likely to experience sickness absenteeism than those who did so. Timely medical examination helps as a preventive medicine for detecting and treating early initiation of health problems on regular basis before it goes to hard step. Thus, it is a moral imperative for the employers to know health status of their employees periodically and take the necessary interventions to protect them from further harming.

In this and other studies employees who worked for greater than 48 h per week were more likely to experience sickness absenteeism than those who did not [5, 19]. This is true for employees who engaged in overtime work [20]. The reason could be that working above normal working hours would affect muscular activities and caused mental fatigue which might lead employees to increased risk of accidents. It could be recommended that assignment of employees should take into account the implementation of shiftwork scheduling. Moreover, recruiting additional employees could also improve the situation.

Job dissatisfaction and stress were also found significantly associated with sickness absenteeism. Other studies support these findings [21–24]. Existing research has recognized heavy workload, insufficient resources, work relationships, lack of professional respect, and lack of promotion opportunities as possibly the most salient job stressors [25–27]. Chronic exposure to stress may be harmful to the health of employees and may also affect productivity of organization through employee dissatisfaction, burnout, poor performance, high turnover, and increased absence from work [28–32].

Social desirability bias is a potential limitation in self-reported studies like this one, in that employees might report more socially acceptable responses than their actual day to day practice. As this is a cross-sectional study, the limitations that come with this type of design need to be taken into consideration when interpreting the findings.

## Conclusions

In this study a relatively higher rate of sickness absenteeism was reported compared to other studies. Interventions to reduce sickness absenteeism should focus on areas, such as periodic medical checkup, monitoring work schedules, improving employees’ job satisfaction, and reducing workplace stress.
